# Enumeration of *Escherichia coli* in Probiotic Products

**DOI:** 10.3390/microorganisms7100437

**Published:** 2019-10-11

**Authors:** Camille Zimmer, Caetano Dorea

**Affiliations:** Department of Civil Engineering, University of Victoria, Victoria, BC V8P 5C2, Canada; caetanodorea@uvic.ca

**Keywords:** probiotics, live biotherapeutics, *Escherichia coli*, viable cell counts

## Abstract

Probiotic products typically take the form of oral supplements or food-based products containing microorganisms, typically bacteria. The number of bacteria present in a dose of probiotic can be several orders of magnitude lower than the label claims, and in some cases, undetectable. The objective of this study was to assess probiotic products containing *Escherichia coli* to verify manufacturer claims, which have not yet been independently assessed, regarding the number of viable *E. coli* per suggested dose. It was found that the products tested contained *E. coli* in numbers several orders of magnitude less than claimed, and when subjected to simulated stomach conditions, the number of viable *E. coli* was significantly reduced.

## 1. Introduction

Numerous commercially available probiotic products have been marketed to consumers using claims of improving digestion and general health. Probiotic products, which come in a variety of forms, including powders, pills, liquid suspensions, and food products, have seen a significant rise in recent years in the number of such products available [1].

Despite recommendations that a probiotic should contain a number of viable cells greater than 10^6^ to 10^8^ to be efficacious [2], studies enumerating viable cells present in a dose (as defined on the label, e.g., drops of liquid, pills, volume, or other unit of powder) of probiotic can be several orders of magnitude lower than manufacturer claims, and in some cases are undetectable [3,4,5,6]. Such studies have assessed common bacterial probiotics, such as *Bifidobacterium*, *Lactobacillus*, and *Streptococci*.

Probiotic products containing *Escherichia coli* have not yet been assessed to verify manufacturer claims regarding number of *E. coli* per dose of supplement. In randomized, controlled, double-blind clinical trials using human subjects, *E. coli*-based probiotics have been effective in reducing symptoms of constipation [7] and inflammatory bowel disease (IBD) [8], and were found to be equally as efficacious as the typical mesalazine treatment in preventing a relapse of ulcerative colitis (UC) [9]. However, several review articles have found conflicting results from multiple studies regarding the efficacy of *E. coli* probiotics in reducing symptoms compared to a placebo or standard treatment [10], with a lack of placebo-control or blinding in some studies [10,11]; these articles have noted that the mechanisms within the gut by which *E. coli* probiotics reduce symptoms is not completely understood [12], and a centralized and systematic approach to investigate *E. coli* products and their properties is lacking [11].

Although instructive, an assessment of respective *E. coli* products for enumeration fell outside the scope of the clinical trials and reviews described above. The objective of this study was to assess probiotic products containing *E. coli*, in order to verify manufacturer claims regarding the number of viable *E. coli* in those products.

## 2. Materials and Methods

### 2.1. Escherichia coli Probiotic Products 

Two brands of *E. coli* probiotic products were assessed for viable *E. coli* enumeration: Mutaflor (Pharma-Zentrale GmbH, Herdecke, Germany) and Symbioflor 2 (SymbioPharm GmbH, Herborn, Germany). Mutaflor claims to contain viable cells of a single *E. coli* strain called *E. coli* Nissle (EcN; DSM 6601) [13,14], and Symbioflor 2 claims to contain a mix of viable cells from six *E. coli* genotypes (DSM 17252) [10,15]. Mutaflor and Symbioflor 2 product samples from three different lot numbers were purchased; once received, samples were kept refrigerated until use. All tests were conducted before stated product expiry dates. Product characteristics are summarised in Table 1. 

### 2.2. Product Testing 

The enumeration of viable *E. coli* in probiotic products was carried out under three test conditions. In Condition 1, quantification of *E. coli* in the supplements was performed after product dissolution in sterilized, buffered, quarter-strength isotonic Ringers (Oxoid Ltd., England, United Kingdom) solution, following an established framework for assessing probiotic products [2]. Whereas test Condition 1 verified viable *E. coli* counts in the products, it did not account for possible effects of acidic stomach conditions, which could affect viability counts. Condition 2 assessed *E. coli* enumeration after the products were to subjected to simulated acidic (pH 2.0) stomach conditions for 3 h, as has been performed in similar evaluations [16,17]. Although mechanical digestion steps such as mastication and churning were not simulated, Mutaflor, being an encapsulated product, was assessed with regard to capsule integrity in simulated acidic stomach conditions (Condition 3). For both products examined, the lot number with the highest *E. coli* count resulting from Condition 1 was selected to be tested a minimum of three times under Condition 2. One Mutaflor capsule from each lot number was examined under Condition 3.

Intact Mutaflor capsules were sterilized by wiping the outside with 70% ethanol (Commercial Alcohols, Brampton, ON, Canada) and allowed to dry before aseptically opening and carefully depositing the powder contents into approximately 100 mL of sterile Ringers solution, warmed to 37 °C [2]. Symbioflor 2 bottle nozzles were sterilized with 70% ethanol and allowed to dry before transferring 1 mL of liquid suspension to 100 mL of sterile Ringers solution. Both mixtures were stirred on a magnetic stirrer on maximum speed (1800 rpm) for 15 min inside a 37 °C incubator [2].

Under Condition 2, the pH of the Ringers suspension containing the probiotic sample was adjusted to 2.0, using a sterile 0.1 N HCl solution (Acculute, VWR, Radnor, PA, United States), then was kept inside a 37 °C incubator, stirring on low (500 rpm) before and after the incubation period. After 3 h, the pH was neutralized to pH 7.0 using a sterile 0.1 N NaOH solution (Bio Basic Inc., Toronto, ON, Canada) [16,17], and the entire neutralized mixture (approximately 100 mL) was enumerated.

In Condition 3, Mutaflor capsules were placed in a pH-adjusted Ringers solution (as in Condition 2) for a 3 h test period. Capsules were visually inspected to determine if breakage occurred on the capsule surface. If a rupture was detected, the Ringers solution was neutralized (as in Condition 2), and *E. coli* was enumerated. 

### 2.3. Analytical Methods

Under Condition 1, 1.0 mL of the suspension was withdrawn, and serial 10-fold dilutions were carried out using sterile Ringers solution. Under Condition 2, 100 mL of neutralized Ringers solution containing the probiotic sample was used for enumeration, without dilution. All samples were enumerated in triplicate using a Colilert Quanti-tray/2000 system, following the manufacturer’s instructions [18]. pH was measured with a digital probe (PHC101, Hach, London, ON, Canada) and multimeter (HQ40d, Hach, London, ON, Canada). The enumeration methods used in this study include only counts of viable *E. coli*.

### 2.4. Statistical Methods

Descriptive statistics were used to characterize *E. coli* counts from samples, including arithmetic and geometric mean values, 95% confidence intervals (CIs) from triplicate trials. Results are graphically displayed, using log_10_-transformed data points for clarity. A value of 0.5 for the most probable number (MPN), half of the method detection limit, was used for data points representing non-detects when calculating means and log_10_ reductions involving non-detects. A one-way analysis of variance (ANOVA) was carried out to compare differences between lot numbers of each probiotic product under Condition 1. Further analysis of the variation between each individual lot number was undertaken using the post-hoc Bonferroni correction, to reduce the likelihood of a Type I error when making multiple comparisons. A Welch two-sample *t*-test was performed to compare the *E. coli* counts between Conditions 1 and 2. Any differences were considered significant at the *p* ≤ 0.05 significance level. All statistical tests were performed using non-log_10_-transformed data, with R statistical software, version 3.4.3. 

## 3. Results

**Figure 1:**
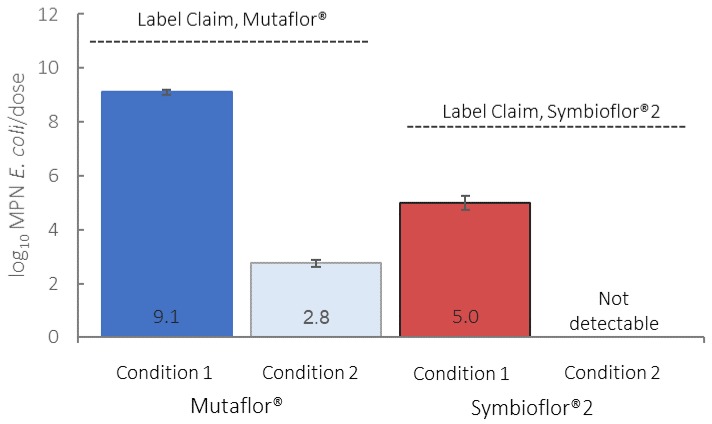
Comparison of log_10_-transformed arithmetic mean *E. coli* counts to manufacturer claims under Conditions 1 and 2; error bars denote a 95% confidence interval (CI).

Under Condition 1, the contents of one capsule of Mutaflor yielded an arithmetic mean of 8.5 log_10_ MPN (most probable number) of *E. coli* (95% CI 8.4–8.6), and a geometric mean of 8.1 log_10_ MPN *E. coli* (95% CI 7.5–8.7). The expected arithmetic mean of four capsules together (one dose, as specified on the label) is 9.1 log_10_ MPN *E. coli* (95% CI 9.0–9.2), and the geometric mean per dose is 8.7 log_10_ MPN *E. coli* (95% CI 8.1–9.3). The contents of the 1 mL recommended dose of Symbioflor 2 yielded an arithmetic mean of 5.0 log_10_ MPN *E. coli* (95% CI 4.6–5.2), and the geometric mean is 4.8 log_10_ MPN *E. coli* (95% CI 4.6–4.9). A comparison of products with their respective product labels is shown in Figure 1. *E. coli* numbers in both products were approximately 2 orders of magnitude under the label claims.

Under Condition 2, one capsule from the highest-count Mutaflor probiotic sample yielded an arithmetic mean of 2.1 log_10_ MPN *E. coli* (95% CI < 1–2.4), and a geometric mean of 0.61 log_10_ MPN *E. coli* (95% CI < 1–1.7). The expected arithmetic mean of four capsules together (one dose, as specified on the label) is 2.7 log_10_ MPN *E. coli* (95% CI < 1–3.2), and a geometric mean per dose of 1.2 log_10_ MPN *E. coli* (95% CI < 1–2.3). The contents of the 1 mL recommended dose of the highest-count Symbioflor 2 probiotic sample yielded an *E. coli* count below the method detection limit, indicating that the *E. coli* in Symbioflor 2 likely do not survive the acidic conditions as tested. The counts under Conditions 1 and 2 were statistically significant for both Mutaflor and Symbioflor 2, yielding *p*-values of <0.01. 

An ANOVA revealed significant differences in *E. coli* counts between lot numbers of Mutaflor (*p* < 0.01), but not Symbioflor 2 samples (*p* = 0.06), enumerated under Condition 1 (Table 2). A post-hoc Bonferroni analysis of the variation between Mutaflor^®^ lot numbers showed that the *E. coli* counts in lot numbers 740170 and 810180 were significantly different from each other (*p* < 0.01), by a mean difference of 0.67 log_10_, while the remaining lot numbers tested were not significantly different from each other. 

Under Condition 3, all Mutaflor capsules tested stayed intact. Although this representation does not factor into the mechanical digestion steps, it does indicate the potential for the capsule to survive the acidic conditions of the stomach, as advertised by the manufacturer [13]. It has been noted [1,19,20] that encapsulation and microencapsulation do offer protection against stomach conditions, to give probiotic bacteria a chance to reach the lower gastrointestinal (GI) tract. 

## 4. Discussion

Two *E. coli* probiotics were assessed, and both yielded *E. coli* counts of approximately two orders of magnitude lower than the respective label claims. This is aligned with previous studies regarding discrepancies between the label claims of probiotics and the delivery and retention of probiotic organisms within the lower GI tract [3,4,5,6]. 

Although there is an established framework in the literature [2] to enumerate probiotic products, in the region of study (Canada) there is not currently oversight by the government with respect to methods of probiotic enumeration [21] to obtain label claims, and there is little transparency by manufacturers with respect to their enumeration methods. The manufacturer of Mutaflor cited a plate count method using non-selective Trypic Soy Agar, which does not select for *E. coli* in enumeration; to our knowledge, specific *E. coli* counts were not confirmed by the manufacturer as were examined in this study. Manufacturer methods to enumerate Symbioflor *E. coli* counts were not reported, despite efforts to contact the manufacturer. Due to the enumeration methods used in this study, it was only confirmed that the bacteria contained in both probiotic products were *E. coli*, not that the *E. coli* strains in the products matched any EcN (DSM 6601) or DSM 17252 reference strains. Further analysis, beyond the scope of this study, would be required to compare *E. coli* in the probiotics tested with a reference strain; both EcN (DSM 6601) and DSM 17252 strains are maintained at the Deutsche Sammlung von Mikroorganismen und Zellkulturen (German Collection of Microorganisms and Cell Cultures) (DSMZ; Braunschweig, Germany) [22].

The results above indicate that the *E. coli* in Symbioflor 2 likely do not survive the acidic conditions as tested, and that if the Mutaflor capsule is ruptured during digestion, and the contents exposed to a low-pH environment, the resulting arithmetic mean *E. coli* count is 2.1 log_10_ MPN *E. coli*. By comparison, the mean count of viable *E. coli* in the lower intestine is between 3.3 and 6.2 log_10_ [23]. *E. coli* is not among the 57 most abundant microbiological species found in a study of 124 European individuals [24], potentially limiting the impact of *E. coli* probiotic products on gut health, due to competition from other microbiological species for gut colonization [10]. 

The discrepancy between the general marketing claims of probiotics and actual delivery and retention of bacteria to the lower GI tract have been noted by others [1,3], expressing concerns regarding labelling following findings of erroneous identification of bacterial strains present in probiotics, as well as bacterial strains present that were not mentioned on the label [3,25,26] and instability of the bacterial strains over the shelf life [27]. Further, the mechanisms within the gut by which *E. coli* probiotics reduce symptoms is not completely understood [12], and evidence regarding the adherence of probiotic organisms to epithelial cell walls in the lower GI tract is lacking [28]. In two studies involving human subjects, Mutaflor was given orally; after being administered for one week, EcN could only be detected in 57% of stools one week following ingestion [29]; in another study, after being administered for 17 days, EcN could only be detected in 40% of stools two weeks following ingestion, with the number falling to 20% nine weeks following ingestion [30]. Conflicting evidence regarding the efficacy of *E. coli* probiotics to reduce symptoms in comparison to a placebo or standard treatment [10], as well as a lack of placebo control or blinding in some studies [10,11], has been pointed out by systematic reviews, suggesting the lack of a centralized and systematic approach to investigate *E. coli* products and their properties [11].

Our study adds to the body of studies finding the number of viable strains present in a dose of a given probiotic to be several orders of magnitude lower than label claims, or undetectable [3,4,5,6]. These findings should be used when interpreting the outcomes of trials using such probiotic products. Due to such concerns, it has been recommended by others that consistent methodology be used for the enumeration of probiotic products [2]. 

## Figures and Tables

**Table 1:** Characteristics of studied *E. coli* probiotic products: Mutaflor and Symbioflor 2.

Product Name	Format	Dose ^1^	Manufacturer Claim of *E. coli* Count per Dose ^1^	Lot Number	Purchase Date	Expiry Date ^1^
Mutaflor	Powder inside a capsule	minimum 4 capsules per day	>1.0 × 10^11^ per 4 capsules	730160	10 February 2018	11 August 2018
				74170	10 February 2018	11 August 2018
				810180	21 June 2018	25 January 2019
Symbioflor 2	Liquid suspension	1 mL	1.5–4.5 × 10^7^	2564	14 July 2018	November 2018
				2582	14 July 2018	June 2019
				2584	14 July 2018	August 2019

^1^ As specified on product label.

**Table 2:** Results of ANOVA between the enumeration of lot numbers of Mutaflor and Symbioflor 2 under test Condition 1.

Product Name	Total Degrees of Freedom	*p*-Value
Mutaflor	28	<0.01 ^1^
Symbioflor 2	29	0.06

^1^ Significant results at the *p* ≤ 0.05 confidence level.
